# Health-related quality of life, assessed with a disease-specific questionnaire, in Swedish adults suffering from well-diagnosed food allergy to staple foods

**DOI:** 10.1186/2045-7022-3-21

**Published:** 2013-07-01

**Authors:** Sven-Arne Jansson, Marianne Heibert-Arnlind, Roelinde JM Middelveld, Ulf J Bengtsson, Ann-Charlotte Sundqvist, Ingrid Kallström-Bengtsson, Birgitta Marklund, Georgios Rentzos, Johanna Åkerström, Eva Östblom, Sven-Erik Dahlén, Staffan Ahlstedt

**Affiliations:** 1 The Centre for Allergy Research, Karolinska Institutet, P.O. Box 287, 17177, Stockholm, Sweden; 2 Swedish Council on Health Technology Assessment, SBU, Stockholm, Sweden; 3 Allergy Unit, Sahlgrenska University Hospital, Gothenburg, Sweden; 4 Sachs’ Children and Youth Hospital, Södersjukhuset, Stockholm, Sweden; 5 The Swedish Asthma and Allergy Foundation, Stockholm, Sweden; 6 Department of Health and Caring Sciences, Linnaeus University, Kalmar, Sweden; 7 Department of Learning, Informatics, Management and Ethics, and Medical Management Centre, Karolinska Institutet, Stockholm, Sweden; 8 The Institute of Environmental Medicine, Karolinska Institutet, Stockholm, Sweden; 9 Dept of Clinical Research and Education, Södersjukhuset, Karolinska Institutet, Stockholm, Sweden

**Keywords:** Food allergy, Adults, Health-related quality of life, Instrument, Questionnaire

## Abstract

**Background:**

Our aim was to investigate the factors that affect health related quality of life (HRQL) in adult Swedish food allergic patients objectively diagnosed with allergy to at least one of the staple foods cow’s milk, hen’s egg or wheat. The number of foods involved, the type and severity of symptoms, as well as concomitant allergic disorders were assessed.

**Methods:**

The disease-specific food allergy quality of life questionnaire (FAQLQ-AF), developed within EuroPrevall, was utilized. The questionnaire had four domains: Allergen Avoidance and Dietary Restrictions (AADR), Emotional Impact (EI), Risk of Accidental Exposure (RAE) and Food Allergy related Health (FAH). Comparisons were made with the outcome of the generic questionnaire EuroQol Health Questionnaire, 5 Dimensions (EQ-5D). The patients were recruited at an outpatient allergy clinic, based on a convincing history of food allergy supplemented by analysis of specific IgE to the foods in question. Seventy-nine patients participated (28 males, 51 females, mean-age 41 years).

**Results:**

The domain with the most negative impact on HRQL was AADR, assessing the patients’ experience of dietary restrictions. The domain with the least negative impact on HRQL was FAH, relating to health concerns due to the food allergy. One third of the patients had four concomitant allergic disorders, which had a negative impact on HRQL. Furthermore, asthma in combination with food allergy had a strong impact. Anaphylaxis, and particularly prescription of an epinephrine auto-injector, was associated with low HRQL. These effects were not seen using EQ-5D. Analyses of the symptoms revealed that oral allergy syndrome and cardiovascular symptoms had the greatest impact on HRQL. In contrast, no significant effect on HRQL was seen by the number of food allergies.

**Conclusions:**

The FAQLQ-AF is a valid instrument, and more accurate among patients with allergy to staple foods in comparison to the commonly used generic EQ-5D. It adds important information on HRQL in food allergic adults. We found that the restrictions imposed on the patients due to the diet had the largest negative impact on HRQL. Both severity of the food allergy and the presence of concomitant allergic disorders had a profound impact on HRQL.

## Background

Food allergies affect a substantial proportion of the population. The prevalence of objectively documented IgE-mediated food allergy in adults has been estimated to 1-2% [1-3], whereas the prevalence of self-reported food allergy is much higher (3-35%) [4]. Allergic disorders such as asthma, allergic rhinitis, allergic rhinoconjunctivitis and eczema have a considerable impact on the health-related quality of life (HRQL) of the patients [5-7]. Although living with food allergy has been recognized as troublesome [8] the precise impact of food allergy on HRQL has not been sufficiently elucidated [5].

Previous studies exploring the impact of adverse reactions to food on HRQL in adults have, to our knowledge, used generic rather than disease-specific questionnaires. Disease-specific HRQL questionnaires could be a better tool to elucidate specific factors that could influence HRQL in food allergic patients. A disease-specific HRQL questionnaire focused on food allergy, the Food Allergy Quality of Life Questionnaire (FAQLQ), has recently been developed as part of the EU project EuroPrevall (The Prevalence, Cost and Basis of Food Allergy across Europe) and has been validated for children, adolescents and adults [9-12] as well as for eight different languages (Goossens et al., personal communication). The FAQLQ has been used in European countries and the USA [13].

The aim of the present study was to investigate HRQL in a Swedish adult cohort with well-diagnosed food allergy to the staple foods cow’s milk, hen’s egg or wheat, by using the disease-specific FAQLQ, and to compare these results with the outcome of the generic EuroQol Health Questionnaire, 5 Dimensions (EQ-5D). A second aim of the study was to investigate if we could identify specific factors with impact on HRQL in this population.

## Methods

### Study sample

**Figure 1:**
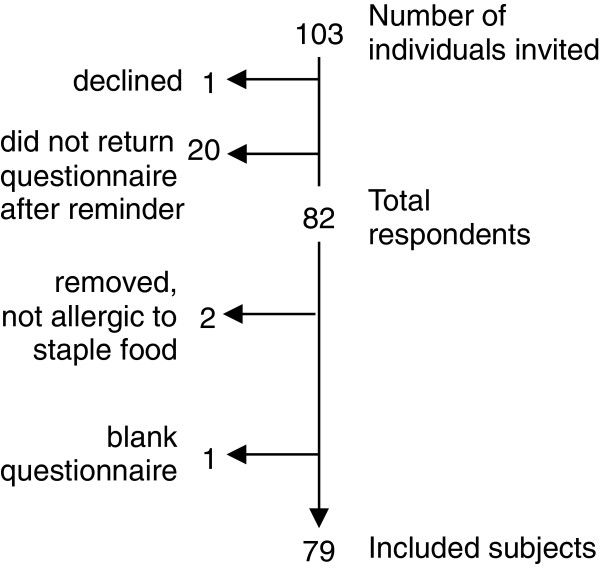
Study flow chart.

The recruited patients had documented allergy to at least one of the staple foods cow’s milk, hen’s egg or wheat. These particular staple foods were selected since they are difficult to avoid in a Swedish every day diet. The study population consisted of patients with a doctor’s diagnosis of food allergy to any of the three above mentioned staple foods, registered at the outpatient allergy clinic at the Sahlgrenska University Hospital in Gothenburg. The patients were identified and recruited by a clinical dietician during 2010 and 2011 based on medical records. Inclusion criteria were a convincing history of food allergy to at least one of the three staple foods either ascertained by a positive food challenge with objective symptoms, or by high levels of food specific IgE antibodies with strong association to positive double-blind food challenge results according to today´s standard procedure [3,14-18]. In total, 103 patients fulfilling the criteria were invited, and received the FAQLQ and EQ-5D questionnaires by regular post together with a formal invitation letter containing information about the study and a pre-paid return envelope. When returning the completed questionnaires an incentive of two movie tickets was offered to the respondents. After one reminder, a total of 80% of the respondents had returned the questionnaires. Two were excluded because the respondents reported not to be allergic to cow’s milk, hen’s egg or wheat any longer, and one person returned a blank questionnaire. Finally, 79 patients (28 males and 51 females) were included in the study (Figure 1). The mean age of the patients was 41 years (range 19–78).

### Food Allergy Quality of Life Questionnaire (FAQLQ)

The FAQLQ is available in four versions; an adult form, a teenager form (13–17 years of age), a child form (8–12 years of age), and a parent form for children (0–12 years of age). In this study, we used the adult form, FAQLQ-AF [19]. The validation of the original FAQLQ-AF was done in the Netherlands where it was found to be valid and reliable, and it was able to discriminate between patients with different disease characteristics [20]. The FAQLQ-AF was subsequently translated to eight other languages (including Swedish) and all versions have recently been validated (Goossens et al., personal communication).

The translation of the FAQLQ-AF questionnaire to Swedish was done according to the guidelines set up by the WHO [21]. Thus, the questionnaire was first translated from English to Swedish by a native Swedish speaker (forward translation), then back-translated into English by a native English speaker, and finally the back-translated version was compared to the original English questionnaire. The translations were done by persons with medical skills. A pilot trial was conducted in 10 patients to ascertain that the translated FAQLQ-AF was firmly understood by Swedish-speaking persons. From the results of the pilot study it was concluded that the translation of the instrument to Swedish worked after a few minor linguistic adjustments.

The FAQLQ-AF assesses HRQL in four domains: Allergen Avoidance and Dietary Restrictions (AADR), Emotional Impact (EI), Risk of Accidental Exposure (RAE), and Food Allergy related Health (FAH), containing a total of 29 items (Table 1).

**Table 1:** **The items in the four domains in the FAQLQ**-**AF questionnaire**

**Allergen Avoidance and Dietary Restrictions (AADR)**	
**How *troublesome *do you find it, because of your food allergy, that you …**	… must always be alert as to what you are eating?
	… are able to eat fewer products?
	… are limited as to the products you can buy?
	… must read labels?
	… must refuse many things during social activities?
	… are less able to spontaneously accept an invitation to stay for a meal?
	… are less able to taste or try various products when eating out?
	… can eat out less?
	… must personally check whether you can eat something
	when eating out?
	… hesitate eating a product when you have doubts about it?
**How *troublesome *is it, because of your food allergy, …**	… that you must explain to those around you that you have a food allergy?
**Emotional Impact (EI)**	
**How *troublesome *do you find it, because of your food allergy, that you …**	… have the feeling that you have less control of what you eat when eating out?
**How *frightened *are you because of your food allergy …**	… of an allergic reaction?
	… of accidentally eating something wrong?
	… of an allergic reaction when eating out despite the fact that your dietary restrictions have been discussed beforehand?
**Answer the following questions:**	To what degree do you *feel you are being a nuisance* because you have a food allergy when eating out?
	How *discouraged* do you feel during an allergic reaction?
	How *apprehensive* are you about eating something you have never eaten before?
**Risk of Accidental Exposure (RAE)**	
**How *troublesome *do you find it, because of your food allergy, that you …**	… sometimes frustrate people when they are making an effort to accommodate your food allergy?
**How *troublesome *is it, because of your food allergy, …**	… that the ingredients of a product change?
	… that labels are incomplete?
	… that the lettering on labels is too small?
	… that the label states: “May contain (traces of)….”?
	… that ingredients are different in other countries (for example during vacation)?
	… that people underestimate your problems caused by food allergy?
	… for your host or hostess should you have an allergic reaction?
**Food Allergy related Health (FAH)**	
**How *troublesome *is it, because of your food allergy, …**	… that it is unclear to which foods you are allergic?
**How *worried *are you because of your food allergy …**	… about your health?
	… that the allergic reactions to foods will become increasingly severe?

The questionnaire scores are based on a 7-point scale, where 1 is the best possible (highest HRQL) and 7 the worst possible score (lowest HRQL) [10,20,22,23]. Mean HRQL scores are analyzed in each of the four domains and a mean total HRQL score is estimated according to the results in the four domains.

### Floor and ceiling effects

Floor and ceiling effects (percentages of patients with minimal and maximal scores, respectively) of the FAQLQ-AF were investigated in order to verify the validity and reliability of its contents. Such effects were considered to be present if more than 15% of the patients in a sample of at least 50 patients achieved the lowest or highest possible scores, respectively. If floor or ceiling effects are present, it is likely that extreme items are missing in the lower or upper end of the scale. In such cases, as a consequence, patients with the lowest or highest possible scores cannot be distinguished from each other, and reliability of the questionnaire is reduced [24].

### Symptoms

Questions regarding the different symptoms that arise as a result of the food intake were included in the FAQLQ-AF. All reported symptoms were aggregated into groups according to the organs that were affected. In the present study, the definition of anaphylaxis includes the four self-reported symptoms “difficulty breathing” and/or “inability to stand”, collapse, loss of consciousness, representing reactions in the respiratory tract and/or cardiovascular system according to definition of anaphylaxis [25-27]. The classification of symptoms is shown in Table 2.

**Table 2:** **Classification of symptoms in the FAQLQ**-**AF**

**Groups of symptoms**	**Symptoms**
Skin	itchy ears, itchy skin, red rash, swelling of the skin, hives, worsening eczema
Oral Allergy Syndrome (OAS)	itchy mouth, itchy throat, itchy tongue, itchy lips
Mucous membrane	swollen tongue, swollen lips, runny nose, blocked nose, sneezing, itchy eyes, watery eyes, red eyes, tightening throat, difficulty swallowing
Respiratory	hoarseness, difficulty breathing, wheezing, cough
Gastrointestinal	nausea, stomach cramps, vomiting, diarrhea
Cardiovascular	dizziness, palpitations, loss of vision, inability to stand, light headedness, collapse, loss of consciousness

### EuroQol Health Questionnaire, five Dimensions (EQ-5D)

The generic EQ-5D health-related quality of life questionnaire was used for comparison to the disease-specific FAQLQ-AF. The EQ-5D was developed by the EuroQol Group [28] and comprises of five dimensions (mobility, self-care, usual activities, pain/discomfort and anxiety/depression). Each dimension has three levels: no problems, some problems, severe problems. The questionnaire responses were converted into a single summary index by applying a formula that attaches values (preference-based weights elicited from population surveys) to each of the levels in each dimension. As there is no Swedish index tariff, for this analysis we used the United Kingdom time trade-off value set based on the UK EQ-5D index tariff [29]. An index of 1.0 corresponds to full health (highest HRQL) and 0 the worst possible score (lowest HRQL).

### Statistics

The statistical analysis was performed with the IBM Statistical Package for the Social Sciences (SPSS) Statistics 20. Parametric one-samples T-test was used to test statistical significance with 0.95 percent confidence interval. Linear regression was used to estimate which symptom groups that had the largest negative impact on HRQL.

### Ethics

The study was approved by the Regional Ethical Review Board in Stockholm, Sweden (Dnr 2009/84-31/5), and the collected personal data was treated according to the Swedish personal data act.

### Consent

Written informed consent was obtained from each patient in this study when responding to the questionnaire for the publication of this report and any accompanying images.

## Results

### Characteristics of the study population

The allergy characteristics of the 79 patients (28 males and 51 females) are shown in Table 3. Briefly, allergies to the staple foods cow’s milk, hen’s egg or wheat were evenly distributed. About half of the patients had a concomitant allergy to tree nuts and/or peanuts. Allergy to shell fish and fish was more common in males, compared to females. About half of the patients had allergy to three or more foods and less than one fifth of the patients had allergy to one or two foods only. Symptoms from the mucous membranes and skin were more common and no differences were seen between men and women, whereas symptoms from the gastrointestinal tract were more common in females. Almost all (90%) of the patients had at least one concomitant allergic disorder (such as asthma, allergic rhino-sinusitis, allergic rhinoconjunctivitis or eczema), and one third were affected by four other allergic disorders in addition to food allergy.

**Table 3:** Descriptive allergy characteristics of the patients

	**Males**	**Females**	**Total**
	**n (%)**	**n (%)**	**n (%)**
*Offending staple foods*, *inclusion criteria*			
Cow’s milk	19 (68)	27 (53)	46 (58)
Wheat	15 (54)	25 (49)	40 (51)
Hen’s egg	14 (50)	25 (49)	38 (48)
*Other offending foods reported by patients*			
Tree nuts	13 (46)	29 (57)	42 (53)
Peanuts	14 (50)	24 (47)	38 (48)
Fruit	11 (39)	22 (43)	33 (42)
Shell fish	12 (43)	14 (27)	26 (33)
Vegetables	8 (29)	15 (29)	23 (29)
Fish	9 (32)	9 (18)	18 (23)
Soy	6 (21)	12 (24)	18 (23)
Celery	7 (25)	4 (8)	11 (14)
Sesame seed	5 (18)	3 (6)	8 (10)
*Number of offending food items*			
1 food	6 (21)	8 (16)	14 (18)
2 foods	6 (21)	6 (12)	12 (15)
3 foods	1 (4	8 (16)	9 (11)
>3 foods	29 (57)	44 (56)	44 (56)
*Type of symptoms*			
Skin	20 (71)	38 (75)	58 (73)
Oral Allergy Syndrome	15 (54)	35 (69)	50 (63)
Mucous membrane	22 (79)	40 (78)	62 (78)
Respiratory	18 (64)	31 (61)	49 (62)
Gastrointestinal	15 (54)	41 (80)	56 (71)
Cardiovascular	15 (54)	26 (51)	41 (52)
*Number of concomitant allergic disorders*			
0 allergic disorder	4 (14)	5 (10)	9 (11)
1 allergic disorder	5 (18)	4 (8)	9 (11)
2 allergic disorders	7 (25)	9 (18)	16 (20)
3 allergic disorders	4 (14)	15 (29)	19 (24)
4 allergic disorders	8 (29)	18 (35)	26 (33)

### Floor and ceiling effects

Only few food allergic patients reported the minimal (highest HRQL) or maximal (lowest HRQL) score in each of the domains in the questionnaire. Thus, the analysis showed minimal floor or ceiling effects, which confirms the internal validity of the questionnaire (Table 4).

**Table 4:** **Percentage of floor and ceiling effects in each of the domains of the FAQLQ**-**AF**

	**Floor**	**Ceiling**
	**N (%)**	**N (%)**
**Allergen Avoidance and Dietary Restrictions** (**AADR**)	0 (0)	6 (7.6)
**Emotional Impact** (**EI**)	0 (0)	5 (6.3)
**Risk of Accidental Exposure** (**RAE**)	0 (0)	1 (1.3)
**Food Allergy related Health** (**FAH**)	5 (6.3)	0 (0)
**Health Related Quality of Life** (**HRQL**)	0 (0)	0 (0)

### Health-related quality of life

**Figure 2:**
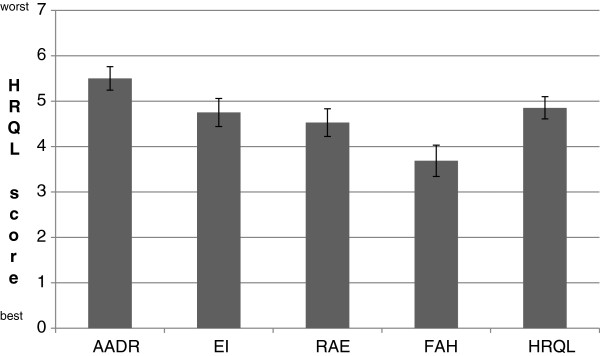
***Domains of the FAQLQ*-*AF (mean score and confidence interval) in food allergic adults.*** The FAQLQ-AF scores were based on a 7-point scale, where 1 is the best possible score (highest HRQL). The questions were divided into the four domains: Allergen Avoidance and Dietary Restrictions (AADR), Emotional Impact (EI), Risk of Accidental Exposure (RAE) and Food Allergy related Health (FAH). Based on the results in the four domains, the overall HRQL was estimated.

The scores in the four domains of the FAQLQ-AF are shown in Figure 2. The highest score (lowest HRQL) was found in the domain AADR (i.e. How troublesome do you find it, because of your food allergy, that you …), and the lowest score (highest HRQL) was found in the domain FAH (i.e. How worried are you because of your food allergy …). The mean HRQL score was estimated to 4.85 (95% CI, 4.61-5.10). No significant difference was found in the mean HRQL scores between males and females (4.81 vs. 4.88, respectively).

### HRQL and concomitant allergic disorders

The presence of concomitant allergic disorders had a profound effect on HRQL in food allergic patients. Patients who, in addition to food allergy, were affected by four other allergic disorders (asthma, allergic rhinitis, allergic rhinoconjunctivitis, eczema and/or other allergic skin problems) reported a lower HRQL compared to those who were affected by three or fewer other allergic disorders (5.21 vs. 4.68; p = 0.03). Moreover, having asthma together with food allergy seemed to have a further profound effect on HRQL compared to patients with food allergy but no asthma (5.03 vs. 4.24; p = 0.01). This is in contrast to the results found in the generic EQ-5D questionnaire, where no difference was seen between patients with and without asthma (mean EQ-5D index value 0.80 vs. 0.79, respectively). Other concomitant allergic diseases did not further affect HRQL negatively, irrespectively of one, two or three concomitant allergic disorders.

### HRQL and severity of food allergy

The severity of food allergy had an impact on HRQL. Severe food allergy was defined as having a prescription for an epinephrine auto injector (EAI), or self-reported previous episodes of anaphylaxis (i.e. the symptoms “difficulty breathing”, “inability to stand”, collapse and/or loss of consciousness). We found that the total FAQLQ-AF scores were higher in patients who were prescribed an EAI (n = 40), which shows that they had lower HRQL compared to patients who did not have an EAI (n = 39) (5.12 vs. 4.58; p = 0.03). However, such a difference was not found when analyzing the results of the EQ-5D questionnaire (mean EQ-5D index values 0.85 vs. 0.75, respectively). The FAQLQ-AF scores among patients who reported anaphylactic reactions (n = 43) compared to those who did not (n = 36), did not reach statistical significance, although there was a tendency (5.04 vs. 4.63 p = 0.10). When using EQ-5D, no differences were found between patients with and without self-reported anaphylaxis, (mean EQ-5D index values 0.81 vs. 0.79, respectively). The number of offending foods (four or more as compared to three or fewer) did not significantly affect the total FAQLQ-AF score (5.01 vs. 4.67; p = 0.18).

**Figure 3:**
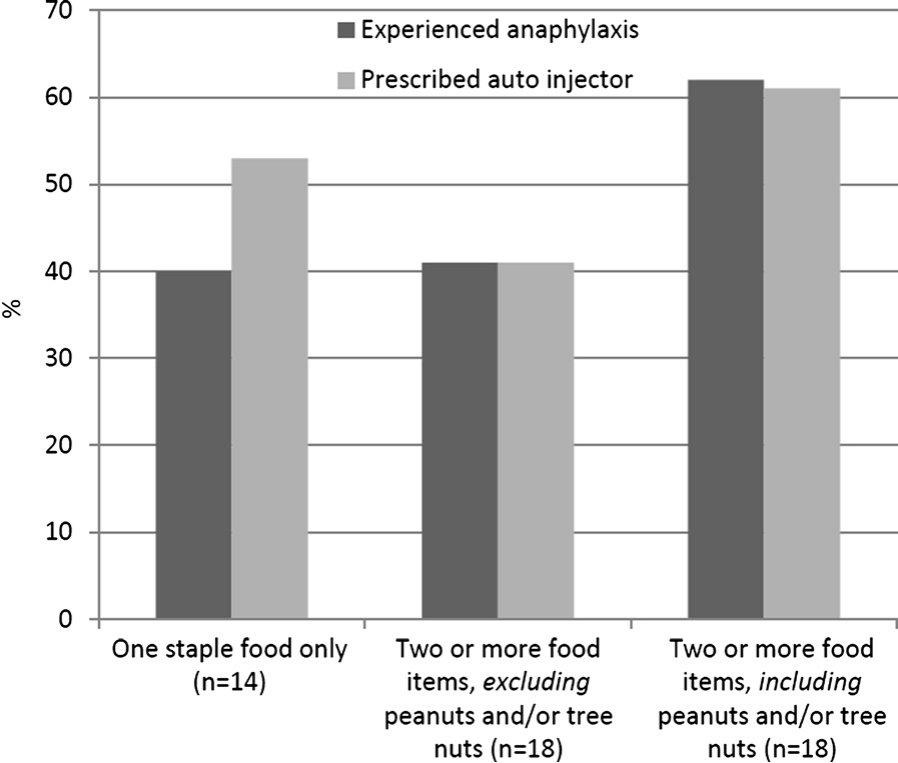
**Distribution of anaphylaxis and prescription of epinephrine auto-injector (EAI) in patients with different types of food allergy.**

Among the 14 individuals who were allergic to only one staple food, 40% had self-reported symptoms of anaphylaxis. The anaphylaxis diagnosis was subsequently verified from the patients’ medical records. However, there were too few patients for further analyses of each individual allergen. In patients allergic to multiple food items but with no allergy to peanuts and/or tree nuts, 41% reported that they had had anaphylaxis. The corresponding figure for patients with multiple food allergies including allergy to peanuts and/or tree nuts was 62%. The distribution of anaphylaxis and prescription of EAI in the three different groups was as presented in Figure 3. Sixty-five percent of the patients with report of respiratory symptoms and 75% of patients with report of cardiovascular symptoms had an EAI prescription.

### HRQL and symptoms of food allergy

The effects of symptoms on the HRQL scores were further analyzed with linear regression. All symptoms were grouped according to the affected organs (Table 3). Symptoms from the skin were reported in 73%, OAS in 63%, mucous membrane in 78%, respiratory in 62%, gastrointestinal in 71% and cardiovascular symptoms in 52% of the patients (Table 3). HRQL was the dependent variable while the symptom groups were independent variables in the regression model. The F-test was significant, indicating a linear relationship between the symptom groups and the HRQL scores and 22% of the variation in the scores could be explained by the symptom groups (R^2^-value of 0.28; (adjusted R^2^of 0.22). The regression coefficients were significant for the OAS and cardiovascular symptom groups, however not for the other symptom groups (Table 5). As HRQL decreases with higher points on the scale, the regression analysis demonstrates that OAS and cardiovascular are the symptom groups with most negative effects on HRQL.

**Table 5:** Effects by symptom groups on HRQL as assessed by linear regression analysis

	**OAS**	**Cardio**	**Gastro**	**Mucous membrane**	**Respir**	**Skin**
**N**	51	41	56	63	49	59
**Mean**	5.13	5.25	4.98	4.93	4.96	4.88
**Regression coefficient**	0.869*	0.754*	0.385	0.057	−0.049	−0.451
**Standardized beta**	0.363	0.328	0.153	0.020	−0.021	−0.172

* Significant, p < 0.05

Concomitant allergic disorders were common among patients experiencing most of the symptom groups (mean of 2.5-2.8 other disorders). However, patients with respiratory symptoms had only 1.8 other allergic disorders on average. The mean numbers of offending food items were quite similar in the different symptom groups (4.2-5.0 food items) (results not shown).

## Discussion

In this study we showed that the food allergy-specific quality of life questionnaire FAQLQ is a valid instrument to investigate health-related quality of life (HRQL) in adult food allergic patients in Sweden. In this cohort with well-diagnosed food allergy to the staple foods cow’s milk, hen’s egg and wheat, we found that the restrictions imposed on the patients due to following a diet had the largest negative effect on HRQL. In addition, both severity of the food allergy and the presence of concomitant allergic disorders had a profound impact on the HRQL of these patients.

Only a few studies in adults have so far been published using a disease-specific validated questionnaire for food allergy [9], and no such studies have been carried out in Sweden. Since staple foods are difficult to exclude from the diet and accidental exposure is likely, we hypothesized that adhering to a diet excluding the staple foods cow’s milk, hen’s egg or wheat is more likely to negatively impact HRQL than a diet excluding less common foods. We therefore recruited patients with a well-diagnosed allergy to one or more of the above mentioned staple foods in order to study HRQL using the newly developed FAQLQ. A subgroup of these patients had already been included in the analysis by Goossens et al. and were found to have a lower total HRQL score compared to patients in Iceland, Poland, France, Spain, Italy and Greece (personal communication). In addition, we studied the factors that influence HRQL, and we compared the efficacy of the FAQLQ with that of the generic EQ-5D questionnaire. In contrast to the disease-specific FAQLQ-AF, the EQ-5D revealed neither a difference between patients with and without asthma nor between patients with and without anaphylaxis (assessed as a prescription of an EAI). This firmly indicates a better precision of the newly developed disease-specific instrument compared to the generic one.

The domain Allergen Avoidance and Dietary Restrictions (AADR) had the highest score (lowest HRQL) compared to the three other domains, indicating that this domain had the highest impact on HRQL. Furthermore, patients with four concomitant allergic disorders reported significantly lower HRQL compared to patients with three or fewer concomitant allergic disorders. Particularly, patients who had both food allergy and asthma reported a lower HRQL compared to food allergic patients without asthma. Similarly, those having been prescribed an EAI had lower HRQL compared to those who had not, indicating that those with a more severe disease had a lower HRQL. On the other hand, we found that the number of food items that the patient does not tolerate, does not affect HRQL, as patients who were allergic to four or more food items did not have significantly different HRQL compared to patients who were allergic to three or fewer food items. The regression analysis further emphasized that cardiovascular symptoms and symptoms from the respiratory tract caused by the food allergy, especially OAS, had most impact on HRQL. However, it should be noted that some of the patients could have misinterpreted the OAS symptoms for the first signs of airway obstruction and anaphylaxis in such cases where they had experienced these before.

Those with allergy to only one staple food, 36 percent (5 out of 14) reported anaphylactic symptoms (a result that was subsequently verified by the medical records) and 50 percent (7 out of 14) had an EAI prescribed. Whether these results indicate under-reporting of anaphylactic symptoms or over-prescription of auto injectors is not known, and will be of interest to follow-up in future studies.

Our study shows that the FAQLQ-AF questionnaire is a valid instrument for measuring HRQL in food allergic adult patients in Sweden. Use of this instrument also identifies the most important factors that affect HRQL. Furthermore, the lack of minimal floor and ceiling effects indicates robust internal validity. In addition, according to the measurement minimal clinically important difference (MCID) a minimum difference score of 0.5 in a HRQL questionnaire with a 7-point scale has been indicated as clinically meaningful [30,31]. Thus, the lower HRQL found in patients who, apart from food allergy also had four other allergic disorders, asthma or had been prescribed an EAI indicate clinical relevance (MCID scores of 0.53, 0.79 and 0.54 respectively). Such statistical difference conveying a clinical effect was also previously reported for patients with food induced anaphylaxis [20].

Very little is known about the social and economic consequences of food allergy. The current study has shed new light on the impact that food allergy has on the quality of life of patients. This is valuable new information that may help health care professionals and policy makers to develop tools that could lead to better care for patients with food allergy. A better understanding of HRQL may also contribute to a better understanding of the issues related to food allergy within many sectors of society, for instance food producers and suppliers, and may contribute to better legislation. In addition to social impact, food allergy is likely to give rise to increased costs for the patients, their families as well as society [12,32], but this has so far been very little studied. This highlights the need for further analyses of the socio-economic burden of living with food allergy.

## Conclusions

The disease-specific questionnaire FAQLQ-AF is a valid instrument and gives important information when studying quality of life in food allergic adults in Sweden. In particular, the restrictions imposed on the patients due to following a diet was found to be a factor that has a negative impact on HRQL. Moreover, we found that suffering from four concomitant allergic disorders, having asthma together with the food allergy, as well as being prescribed an epinephrine auto-injector also had a negative effect on HRQL. In contrast, HRQL was not affected by the number of offending food items. The new FAQLQ instrument adds new and more precise information on HRQL in food allergic patients, and may be a more accurate instrument in this patient group than the commonly used generic EQ-5D.

## Abbreviations

AADR: Allergen avoidance and Dietary restrictions; EAI: Epinephrine auto injector; EI: Emotional impact; EuroPrevall: The prevalence, Cost and Basis of Food Allergy across Europe; EQ-5D: EuroQol Health Questionnaire, 5 Dimensions; FAH: Food Allergy related Health; FAQLQ: Food Allergy Quality of Life Questionnaire; FAQLQ-AF: Food Allergy Quality of Life Questionnaire, Adult Form; HRQL: Health Related Quality of Life; OAS: Oral allergy syndrome; RAE: Risk of accidental exposure; SPSS: Statistical Package for the Social Sciences.

## Competing interests

The authors declare that they have no competing interests.

## Authors’ contributions

SA, SAJ, MHA, RM and SED initiated and conceived the study. All authors made substantial contributions to conception, planning and design. SAJ and MHA were the main drivers of the pilot study preceding the present one and translated the questionnaire to fit Swedish conditions, together with UB, JÅ, BM and EÖ. JÅ, UB and GR identified, recruited the patients and validated their disease. JÅ, UB, GR, IKB, ACS and RM carried out the data acquisition and cleaning. SAJ carried out the analysis and statistical evaluation of the data. All co-authors made valid contributions when the results were interpreted and formulated. SAJ and MHA drafted the manuscript and shared first authorship. SA and RM revised the manuscript critically for important intellectual content. All authors scrutinized and approved the final manuscript.
